# A qualitative study of the learning processes in young physicians treating suicidal patients: from insecurity to personal pattern knowledge and self-confidence

**DOI:** 10.1186/1472-6920-7-21

**Published:** 2007-07-06

**Authors:** Tordis Sørensen Høifødt, Anne-Grethe Talseth, Reidun Olstad

**Affiliations:** 1 Department of Psychiatric Research and Development, University Hospital of Northern Norway, Åsgård, N-9291 Tromsø, Norway; 2 Tromsø College, Faculty of Health Sciences, M.H. bygget, Breivika, 9293 Tromsø, Norway; 3 Department of Clinical Psychiatry, Institute of Clinical Medicine, University of Tromsø, Åsgård, N-9291 Tromsø, Norway

## Abstract

**Background:**

Little empirical work has been done in studying learning processes among newly educated physicians in the mental health field.

The aim of the study was to shed light on the meaning of newly educated physicians' lived experiences of learning processes related to treating suicidal patients.

**Methods:**

Thirteen newly educated physicians narrated their learning experiences while treating suicidal patients in their own practice. The interview texts were transcribed and interpreted using a phenomenological-hermeneutical method inspired by Ricoeur's philosophy.

**Results:**

There was one main theme, four themes and eleven sub themes. The main theme was: Being in a transitional learning process. The themes and sub themes were: Preparing for practice (Getting tools and training skills, Becoming aware of one's own attitudes); Gaining experience from treating patients (Treating and following up patients over time, Storing memories and recognizing similarities and differences in patients); Participating in the professional community (Being an apprentice, Relating clinical stories and receiving feedback, Sharing emotions from clinical experiences, Receiving support from peers); and Developing personal competence (Having unarticulated awareness, Having emotional knowledge, Achieving self-confidence). The informants gave a detailed account of the learning process; from recognising similarities and differences in patients they have treated, to accumulating pattern knowledge, which then contributed to their personal feelings of competence and confidence. They described their personal competence with cognitive and emotional elements consisting of both articulated and less articulated knowledge. The findings are interpreted in relation to different learning theories that focus on both individual factors and the interaction with the learning environment.

**Conclusion:**

This study provides additional information about learning experiences of young physicians during the critical transition phase from medical school to early professional life. Peers are used for both learning and support and might represent a more powerful resource in the learning process than previously recognized. Emotional experiences do not seem to be adequately focused upon in supervision, which obviously has relevance both for learning and for the well-being of young professionals. The study indicates some areas of the educational system that could profitably be expanded including stimulating more systematically to critical reflection on and in practice, attention to feelings in the reflective process and provision of more performance feedback to young physicians.

## Background

"Learning can be thought of as a process by which behaviour changes as a result of experience" [1]; this is one of many definitions of the concept of learning. In the present study lived experiences of young physicians as to learning in the mental health field is focused. A broad theoretical perspective seems relevant. The need for medical educators to become conversant with different learning theories in order to create appropriate learning environments and optimize learning has been emphasised [2]. Five main theories will be shortly presented: behaviourist, cognitivist, humanist, social learning and constructivist orientations. These are the five theories mostly used in the literature about medical education [2].

The behaviourist model involves a teacher centred approach where the educator's role is to design the environment for the learner that will then give a specific response and behavioural change will occur [1,3]. In medicine the behaviourist approach is frequently used in development and evaluation of clinical skills instruction and simulated case scenarios [2]. However, higher order-activities, strategic behaviour, problem solving and selective attention can not be dealt with adequately in terms of the behaviourist perspective of stimulus-response [3].

Cognitively oriented explanations of learning encompass a wide range of topics with the shared focus upon the internal mental processes within the learner's control [1]. In medical education application of cognitivist learning theory includes, for instance, the construction of concept maps and the development of critical and reflective thinking [2].

Humanist theories consider learning from the perspectives of the human potential for growth [1]. The focus of learning is on the individual learner's needs, self-development and the underlying assumption that the learners take the primary responsibility for their own learning. In medicine this perspective may be relevant to technology based and computer-assisted instruction and is also relevant in learning methodologies of problem-based or situation-based learning that emphasize self-directedness and self-evaluation [2].

Social learning orientation combines elements from both a behaviourist and cognitivist approach and the locus of learning is in the interaction between the person, the learning environment and the desired behaviour [1]. The application of social learning theory to medical education includes demonstration of skills that is followed by guided practice with feedback and opportunities to reflect on the learning, role-modelling and mentoring [2].

The constructivist orientation includes many different related traditions, including social constructivism, socio-cultural perspectives, cultural-historical-activity theory, situated learning and expansive learning. Emphasis is placed on the influence of the social context [3]. Knowledge is not an individual possession but socially shared and emerges from participation in socio-cultural activities. The constructivist orientation supports educational strategies such as the writing of practice narratives, the development of course portfolios, participation in a professional community and exploration of learning experiences in groups providing meaning to daily practice [4]. When sensitivity to context and access to an overall picture of the situation is crucial, as work within multidisciplinary teams, or multicultural settings, this approach might have special advantages [4].

Little empirical work has been done in studying learning processes among newly educated physicians in the mental health field. In an earlier quantitative study; the relationships among the newly educated physicians' background, their attitudes towards psychiatry, some aspects of the learning environment, psychiatric competence and psychiatric self-confidence were explored and a comprehensive empirical model of the interrelationship between the various aspects of the learning process was developed [5]. The present study was designed to supplement this quantitative study with more in-depth qualitative data. It was considered fruitful to study learning processes in detail related to a specific clinical situation.

Treating suicidal patients represent one of the most challenging and complex clinical situations for young physicians. In an already published qualitative study, thirteen newly educated physicians narrated their experiences with suicidal patients. They clearly described their challenges relating to patients, intervening competently, but also the variety of emotional and ethical dilemmas that arose in treating such patients [6]. The same informants were also asked about their learning experiences related to treating suicidal patients and the results from this part constitute the present study.

### The aim of the study

The aim of the study is to shed light on the meaning of newly educated physicians' lived experiences of learning processes related to treating suicidal patients.

## Methods

### Design

The study was qualitative in design and used a phenomenological-hermeneutical method.

### Subjects

The informants in the study graduated from the faculty of medicine at the University of Tromsø, Norway in 2000. Their psychiatric education was divided into two courses over a six-year program. The first was given during the third year, lasted seven weeks and was primarily based on lectures. During their last year of study the students had a four week psychiatric clerkship at the University Hospital. During that clerkship they attended a two day practically oriented suicide intervention workshop, that was based on small group discussions and role-playing [7].

An 18-month internship, including internal medicine, surgery and primary health care, follows graduation from medical school and precedes full authorization as a medical practitioner in Norway. The informants had completed their internship during the last six months prior to the interview. As this study was part of a project on the development of psychiatric competence among medical students, an invitation to participate in interviews, together with questionnaires for addressing other aspects, was sent to the 54 medical candidates [5]. Forty-four responded (81%) anonymously to the questionnaires and 16 of these agreed to participate in in-depth interviews. The intention was to interview all 16, but three could not be interviewed for practical reasons. The informants were seven men and six women; all about 30 years of age. The interviewees were not different from all those eligible for participation in gender and age. At the time of the interview nine of the 13 informants were working in general practice, the remaining in hospitals, but none of them were currently working in mental health services.

### Ethics

Informants received written information about the project and gave informed consent. They were guaranteed confidentiality and anonymity in the publications of the results. The Regional Ethics Committee and the Norwegian Data Protection Agency approved the study.

### The interviews

The first author conducted all the narrative interviews [8] during the first six months of 2002. The interviews lasted 90 to 120 minutes and were audio-taped and transcribed verbatim by the first author. The informants were asked to narrate a story from treating suicidal patients in their own practice [6]. Then they were asked to reflect on what had been the important contributors to their learning related to this clinical situation. The second part of the interviews; focusing the learning experiences of the young physicians is presented in this study.

The interviewer had developed a semi-structured interview guide where topics such as attitudes, knowledge, skills, literature, clinical teaching, supervision and experiences from life in general were listed (See additional file 1). The interview guide was used as a check list for the interviewer, but the informants were asked to relate their learning experiences as freely as possible. The interviewer used clarifying questions and invited the informants to elaborate sequences that were unclear. The questions were intended to assist the informants to continue their account, which in turn, led to a new phase of questions. The purpose of the narrations was to make the experiences explicit and through interpretation and provide meaning to what was being talked about.

### Data analysis

The hermeneutic approach presupposes that the researcher's interpretation and understanding is based on his/her pre-understanding [9,10]. The unconscious part of the researcher's pre-understanding arises, in part, from his or her own culture, language, history, which, though taken for granted, influences the interpretation of the text [10]. This subjectivity can be counteracted by the application of a strict method of interpretation [11].

No specific theoretical perspective was forced upon the interview text, but the chosen theoretical perspectives illuminate the interview text and the interview text illuminates the chosen theories. Sometimes it is requisite with several theories to shed light on various aspects of the interview text [12]. The theories in this way are part of the author's pre-understanding, which is actively used in formulating of the critical comprehension and discussion of the text.

The narrative interviews have been interpreted using a phenomenological-hermeneutic approach inspired by Ricoeur [11,13] which has previously been applied in a similar way [14,15]. This method focuses upon understanding and interpreting the meaning of phenomena of life experiences. Ricoeur [11,13] describes interpretation of a text as a dialectic process from understanding to explanation and then from explanation to critical comprehension. The interpretation includes three phases, naïve reading, structural analysis and critical comprehension. The interpretation was conducted in the following way: The two first authors read and re-reading the interviews. Then the first author developed the meaning of what the text said semantically, and what possible meanings it represented. These possibilities where then further reflected on and put into the context. At the same time, in the interpretation of the text, each of the informants was remembered and the informants were "present" as a silent party in the interpretation of the interview. The second author followed the first author's thoughts and asked questions about what had been done and why. Through this process the first author's mind was opened up to possible and alternative interpretations of findings, and made sure that the findings were grounded in the data [13]. The interview text was examined in order to explain it, and was divided into meaning units, condensed and organized into themes. In the third phase, the text was again considered as a whole while taking into account the research questions, the researcher's pre-understanding, the theoretical perspectives and the structured analysis. A critical comprehension was then formulated and reflected upon. According to Ricoeur [11] a text has multiple but not infinite meanings, and can thus be interpreted in various ways. The present interpretation is only one of several possibilities.

## Results

### Naive understanding

The informants described their learning process during their first two years of practice. Their learning experiences were related to the treating of suicidal patients. However, the informants seemed to reflect on their learning in a more general perspective. The informants reported few memories from medical school and from text books. They found that it was more difficult to grasp psychiatric theory than theory in other medical fields, without relating it to actual patients. However, they did find training specific communication and clinical skills as a useful preparation for practice. The informants emphasized the importance of treating many patients and described how they remembered the stories of the different patients. Some experienced a very supportive professional community and how they had been able to discuss difficult problems in a multidisciplinary group. Other informants experienced that their colleagues did not provide the support they felt they needed.

The newly educated physicians described the process of acquisition of a personal knowledge base and some confidence. They told about the development from insecurity in dealing with a suicidal patient to one in which they had more self-confidence. They described a personal knowledge base that consisted of "gut feelings", pattern knowledge from stored memories and emotional components.

### Structural analysis

The structural analysis results in eleven sub themes, four themes and one main theme, as shown in the table 1.

**Table 1:** Results of structural analysis identifying sub themes and themes related to main theme

**Main theme**: Being in a transitional learning process	
**Themes**	**Sub themes**

1. Preparing for practice	Getting tools and training skills
	Becoming aware of one's own attitudes
2. Gaining experience from treating patients	Treating and following up patients over time
	Storing memories and recognizing similarities and differences in patients
3. Participating in the professional community	Being an apprentice
	Relating clinical stories and receiving feedback
	Sharing emotions from clinical experiences
	Receiving support from peers
4. Developing personal competence	Having an unarticulated awareness
	Having emotional knowledge
	Achieving self-confidence

#### 1. Preparing for practice

##### Getting tools and training skills

Many of the informants emphasized the importance of learning concrete procedures as in the suicide intervention workshop in medical school; how to assess suicidality, and specifically how to ask the patients direct questions about suicidal thoughts and plans. The informants reported that it was extremely important in the beginning to have treatment plans and procedures. One interviewee said:

*It was some kind of a recipe, using several questions and in a way, developing a score for moderate and high risk*.

They referred to practicing by role-playing, how to relate to a patient, how to phrase their questions and deal with different types of feelings that emerge in such clinical situations. One informant told:

*I think it is very important to have the opportunity to role-play those types of situations...Some do not like role-playing and sneer at it, but it is incredible how one can identify with the situation. I think it is very important to play both the role of the suicidal person and the helper, to attempt to get into the mood of a person who is thinking about committing suicide*.

##### Becoming aware of one's own attitudes

The informants reported the importance of being challenged about their own attitudes regarding suicide as occurred during the suicide intervention workshop. Some experienced that they changed their attitudes, from considering suicide as a private matter of little concern to physicians; to becoming aware of their responsibility towards these patients. One interviewee said:

*This was really a "wake-up" call for me. It really changed a lot of my thoughts about suicide...I got direct feedback about my attitudes.... I thought suicide was a private matter. If people wanted to commit suicide, they had to decide for themselves, but afterwards I found that I had to think differently...There are a lot of things I can do to prevent suicide*.

#### 2. Gaining experience from treating patients

##### Treating and following up patients over time

The informants emphasized the importance of learning psychiatry from the practical experience of meeting patients in real life situations. They referred to other medical fields and found psychiatry exceptional as that theory was more difficult to grasp without being able to relate it to real patients. To have dealt with many patients was often considered important to the learning process and provided the young physician with an ability to deal with very different situations. One interviewee said:

*First of all, that many patients in psychiatry, as in surgery, are in a difficult situation which you can follow to see how things [their condition] develop, is very important*.

When young physicians got the opportunity to make independent assessments, to have responsibility and to follow patients over time, it was easier for them to understand the patients' situation. One informant stated:

*To meet patients, is not only taking a short story, but it is meeting them over and over again in an everyday way, to understand how they function [in daily life] is essential*.

##### Storing memories and recognizing similarities and differences in patients

The informants told how they had a memory store of patients in their minds, with both cognitive and affective elements. This was as if they had acquired their own "history databank". When sitting with a new patient, different images of former patients passed before them. They compared and recognized general as well as special aspects of the situation and used these in their assessments of suicidality. One informant said:

*You have a lot of pictures in your head, examples, and you can more specifically feel what parts to use to make a good assessment...To find some reason that is logical, using my own cases, which I have in my head...then create a picture of this suicidal patient one is facing,...meetings are never the same*.

#### 3. Participating in the professional community

##### Being an apprentice

The physicians express the usefulness of working in interdisciplinary teams and together with more experienced professionals. One informant expressed:

*It is invaluable support to have people around you, who know these things. We had a very skilled homecare nurse who knew everybody and exactly what the problem was*.

The informants expressed the need for seniors to talk to and learn from particularly when facing patients who were difficult to understand and treat, such as those who repeatedly hurt themselves. One informant said:

*My supervisor was very important – especially her way of presenting the group of young people with personality disorders. I had tried to understand, but did not get it. She managed to play that helplessness they show and convey it to others. She set up role playing in her office; that helped me understand what had been a complete chaos in my head*.

The informants reported the benefit of observing senior physicians in daily practice, how they related to the patients and what they laid emphasis on in their assessment. However, these experiences seemed to be infrequent.

##### Relating clinical stories and receiving feedback

The informants expressed that telling their stories about different clinical situations served as a way to reflect on their experiences and make them feel more secure in handling the situation. However, the young physicians had varying experiences; some had many opportunities to relate difficult clinical situations to a senior colleague while others rarely did. One interviewee said:

*To exchange experiences with colleagues has been rewarding, especially when you get to talk to a more experienced colleague and tell about one's own experiences, not necessarily to hear if you did right or wrong, but to describe and go through the situation*.

When they told a story about a patient and the senior physician actively responded; the junior became more confident in her practice. One interviewee said:

*I talked with one of the doctors, described briefly what had happened and got feedback that I had done it in a good way*.

Others only received answers on concrete questions they were posing, but were never invited to tell more about their experiences in the clinical situations. One informant expressed:

*Supervision consisted of only answers and advice*.

Some informants also had the impression that health professionals hesitated to make comments that might be construed as critical. Very few informants reported receiving critical responses related to how they handled clinical situations. One interviewee said:

*We are very kind to each other in the health care system; we have great difficulties in saying that you should probably have done this in another way. ..... I often wonder if I will ever receive feedback, unless something really goes wrong*.

Another informant said:

*With good feedback you receive some kind of critique, what was good, what was bad....... I have had very little of that during medical school and my time as an intern, I think that I have hardly experienced it*.

##### Sharing emotions from clinical experiences

The informants expressed their need to share feelings and to communicate insecurity and doubt about their decisions. Sometimes strong unexpected feelings appear, such as aggression and irritation, towards patients who are in suicidal crisis. Talking to a colleague about a difficult situation or critical incident was experienced as a relief and as important for working through their own reactions. One informant said:

*When such difficult and bad things happen, you have to talk about it. He also felt this was a tough case*.

The importance of sharing the responsibility and burdens with somebody was emphasized. One young physician said:

*I talked to the family physician of the patient afterwards and that was good. We try to have a "get together for those involved" after a critical incident to talk it through*.

The informants also told about experiences when they did not get the emotional support they needed. One expressed it as:

*When you talk about it, it can be aired a little, although I never really get it out enough. I always remain with some reactions. I am sure it would have been good to talk those things through, instead of keeping those feelings within. That [feelings] has never been a topic in my supervision*.

##### Receiving support from peers

The informants seemed to use physicians of their own age and from their own class in medical school as their main resources with which to discuss urgent problems. They used each other to talk through clinical situations, sometimes both before and after the actual event, getting advice and response to their way of handling the situation. They wanted their peers to ask questions as a check so that they felt that they had thought about the essential parts of the problem. Even more they seem to turn to their peers when there has been a critical incident. One said:

*Fortunately, I have had good friends, who have also been colleagues, whom I have been able to talk to. You cannot talk to other friends because of confidentiality, and they do not understand. We were a group of people who had come just as far and we had a lot of supportive talks with each other*.

Another interviewee said:

*We were a fine group of interns. I had especially good contact with one. We talked a lot. She had her issues and I had mine. It was a great source of support*.

#### 4. Developing personal competence

##### Having an unarticulated awareness

The informants described a knowledge that was experienced physically. Some called it a "gut feeling". They described it a little differently, but related it to aspects of the contact with the patient – the content and presentation, the verbal and non-verbal queues. This developed during the contact and was related to how they could make sense of the situation and understand the problem. They used this unarticulated awareness as a factor in their assessment of the clinical situation. The informants also report that the "gut feeling" had changed during their time in practice. One physician said:

*The gut feeling, it is not the same as two years back, it is a bodily response, in a way to use oneself as a guiding light. It can be activated from a situation that one recognizes and feels secure in or one that is not recognized and feels insecure in*.

Another informant said:

*The gut feeling; maybe it is related to how they behave, what impression I get of them, how they appear, if they are desperate, indifferent or if they seem a little apathetic.... it is difficult to describe*.

##### Having emotional knowledge

The informants were concerned about using all of themselves in the role of the physician. Knowledge and understanding of one's own way of reacting made it easier for them to more adequately relate to the patient. One informant said:

*I think that it is very important to think about how you yourself are doing, one's own reactions, not to analyze so much, but to think about my patterns of reacting to what is going on around me.... try to think how I would have felt in a similar situation*.

Some had had personal experiences with depression or a suicidal crisis and considered this to be of importance in their meeting with patients in similar situations. Even if it brought up their own potentially painful experiences, they felt it helped make them more sensitive to these patients. One said:

*I thought that what happened [to me] so many years ago did not mean anything, but I realize that a lot of things are related. I did not think that much about it before....I think I now understand others better*.

Some informants expressed insecurity in relating to emotionally disturbed patients. However, some describe how facing the situation gave them a new personal awareness and they felt more comfortable. One interviewee said:

*Fright is related to knowing too little. I did not know that it was possible to have an everyday talk with a schizophrenic patient. You do not have to behave in another way than you are used to. It is not that difficult to communicate as I thought. The fright and feeling of strangeness disappeared quite quickly*.

Another informant did not manage to feel more confident and actually described an increased feeling of insecurity and fear in meeting seriously mentally ill patients. One informant stated:

*I got scared and did not understand anything. It was so unknown to me. Depression was familiar, but psychosis, that is something else*.

##### Achieving self-confidence

The informants described a process of gaining their own understanding of the patient's situation and acquiring an awareness of clinical patterns. A thorough involvement in the problems of the patient over time was necessary to acquire this perspective. When the young physicians experienced that their way of dealing with the situation was working, their clinical confidence grew. One informant related:

*I could see a pattern and it did not lead to suicide. I found the same things, got a little background and saw relationships. One felt that one could somehow grasp it*.

Being more comfortable in the situation makes the physician go more deeply into the patient's story and explore different ways of handling the situation. One informant reflected on his own development and early practice:

It is so important to take time in the beginning. I even followed them myself to the hospital to be sure they got there. I understand today this was not a proper practice.

The informants described a growing feeling of confidence as to how they met a specific patient and situation. The feeling of confidence seemed to have cognitive and affective aspects, which were interconnected in many ways. One physician reported:

*It is such a big difference in meeting those patients before and now. In the beginning I was scared about the whole situation – felt uncomfortable, hardly able to have a real consultation with these patients...I use more time now and try to really grasp what the story is about. It has been useful to dare to ask, to collect information. It is there that you learn*.

To be able to help a patient in a difficult situation gave a feeling of mastering and competence to the informants who told about the importance of such experiences. One physician said:

*I had one psychiatric patient that I really was satisfied with. It was a woman who had a lot of problems in her life and was very depressed. We established a very good relationship and I referred her to the local psychiatric outpatient clinic. She went for consultations there, but she also came to see me regularly. She really improved and she also saw the results. I thought, "Why did she come to me to talk? What can I do?" When you see that it helps, it gives more confidence*.

One informant summed up what elements that might constitute his feeling of confidence:

*Confidence is to know what to do, the next step, to recognize signs, the situation, pictures and feelings, ones own feelings, and to observe the response of the patient as a way to confirm that something is going right. It is also that you can ask others, to dare to ask, seek help and advice, realizing that you are never alone*.

## Discussion

One main theme, Being in a transitional learning process, and four themes; Preparing for practice, Gaining experience from treating patients, Participating in the professional community and Developing personal competence seemed to run through the learning experiences of the newly educated physicians who were treating suicidal patients.

The young physicians gave a detailed description of their lived learning experiences in dealing with a difficult clinical situation that encompassed both cognitive and emotional aspects. This qualitative data provides information that supplements what can be learned from a more quantitative approach. In this way it serves to broaden the knowledge of real world experiences and theories of learning [5]. The importance of narratives in medical education, both for the purpose of better grasping the medical illness and for the understanding of its meaning in the life story of the patient, has been emphasized by Hunter in the book "Doctors' stories" [16].

The informants only report a few elements from medical school that they have found useful in dealing with suicidal patients. These were the learning of practical ways of handling these situations, the possibility to role-play and becoming aware of their own attitudes. These three elements were part of an interactive workshop [7] using small group discussion of attitudes, providing guidelines for suicide assessment and role-playing followed by feedback. This course was developed on the principles for adult learning [1] and Rothman's action research in which the implementation of researched based knowledge is actually practiced [17]. From a theoretical perspective, both a behaviourist orientation, social learning theory and a constructivist approach seem to be relevant in such a course. We have earlier described how medical students reported a feeling of relief to have received practical tools shortly after completing the suicide intervention workshop [18]. It seems that these first impressions from medical school were sustained two years into practice. Further, the newly educated physicians actually reported using these tools in their own practice and experienced them as useful.

Experience is necessary before physicians can incorporate what they have learnt in medical school into the further development of their clinical skills [19]. The informants emphasized the importance of treating their own patients, the necessity of treating a number of patients and being able to follow them over time. The physicians described how they developed their own "databank of patient histories". This is similar to Schmidt et al [20] report of how students and physicians, when seeing patients with similar symptoms, compile "illness scripts". According to these authors, in addition to "illness scripts", physicians also elaborate a set of lively recollections of specific patients with the same problems. These two representational formats seemed to have synergistic effects. The recollections of former patients are stored in episodic memory, which make them easily accessible [20]. These are elements related to cognitive learning theory. Exposure to many different patients and cases are considered as a crucial factor in developing expertise [20]. It is interesting to notice how the informants described this process; from recognising similarities and differences in patients they have treated, to accumulating pattern knowledge, which then contributed to their personal feelings of competence and confidence.

The newly educated physicians emphasized the learning potential within the professional community that came about through observing doctors and other professionals in action. Role modelling, mentoring and collaborative work are common applications of social learning theory [2]. However, aspects from constructivism and situated learning theory [21] are also relevant. From this perspective the focus is not on the individual learner or in the interaction with others, but that knowledge exists across individuals and within the community. The informants reported the usefulness of sharing clinical stories and reflecting together with a colleague. Through critical reflection practitioners might be able to bridge the theory-practice gap and integrate both aspects in their clinical work. Interestingly, ethical questions were not explicitly addressed by the informants, although there seems to be an unarticulated ethical stance of doing ones best for the patient that ran throughout their dialogues.

The related stories contain clinical reasoning, but also emotions that the young physicians wanted response to. Boud and colleagues [22] emphasized the importance of exploring feelings in the reflective process. When also emotions are given attention in the reflective process, important and sometimes hidden obstacles can be removed [22]. Both from a learning perspective and for the well-being of young professionals it seems important to adequately address the feelings which arise from challenging clinical situations.

In previous studies [5,23] supervision did not have a significant impact on newly educated physicians' self-reported confidence, competence and learning benefit. The quality of the relationship between the supervisor and the trainee and continuity over time are generally considered important factors in supervision [24]. However in these studies, supervision was not explored in further detail as to the relationship, the quality of reflection and response as well as the existence of the possibility to share the supervisee's own emotions during supervision. Some of the young doctors had experienced the fruitfulness of reflecting and receiving performance feedback from senior colleagues, but these events seemed to be rare.

The informants reported how peers were important for support. Peers were easier to approach with a challenging patient story and the young physician's insecurity than were senior colleagues. Studies indicate that peers may be in a position to evaluate each others performance. Anonymous medical students' peer assessments are helpful for fellow students to identify unwanted behaviour [25,26]. Students also gave more meaningful feedback on their own and their peers' attitude and behaviour than did faculty or house staff [26]. The present study indicates that peers are used for both learning and support and might represent a more powerful resource for learning than is generally recognized.

The informants told about the importance of positive experiences and mastering. Bandura views ones personal beliefs of personal efficacy as the most central mechanisms of personal agency [27]. Perceived self-efficacy refers to the beliefs in one's capabilities to organize and execute courses of action required to produce a given attainment [27]. The experience of mastery is considered to be the most important source of efficacy beliefs [28]. These are important elements in social learning theory.

It is interesting to note how the informants described the different components of their learning process, how they related them to a practical clinical situation, their development of personal competence with its cognitive and emotional elements as well as possessing both articulated and less articulated knowledge. Tacit knowledge, by definition, refers to the inarticulate aspects that cannot be taught explicitly and therefore are only to be acquired via direct experience [29]. A clinical teacher needs to be able to articulate knowledge that would normally be tacit for a practitioner. This can facilitate the clinical learning process, but also make tacit knowledge more explicit and accessible for conceptual change. The quality of the "gut feeling" can thus be validated and refined. These principles are seldom described and elaborated in the field of psychiatry.

### Methodological considerations

Those who accepted to be interviewed did not differ in age and gender from others in the group of newly educated physicians. However, they could well represent a group of physicians that are more interested and positive, reflective or, perhaps, more personally involved in the issues surrounding suicide. However, their reports seemed to represent experiences typical for young physicians. They were also congruent with findings from previous research, which lends them some additional credibility [30]. The informants talked about situations in the past and reconstructed what happened. The narration of personal experiences has been found to be a useful method previously [31]. In addition, two of the study's authors have independently analyzed and interpreted the data. Nonetheless, the text could well have multiple meanings and be interpreted in various ways. The present interpretation seems credible, yet remains but one of several possibilities [11]. Phenomenological – hermeneutic interpretation should not be seen as factual knowledge, but rather be regarded as contributing to the discourse about the topic at hand [32].

The strengths of this study are the thorough examination of in-depth interviews that addressed both the behavioural, cognitive and emotional aspects of the young physicians' experiences. The considerable and consistent detail in the data also supports the view that there is high content validity in the material [33]. The narratives consistently illustrated the complex interplay between cognition, emotion and action [34].

### Implications for practice

The study indicates some areas with potential for more extensive and comprehensive learning both in medical school and during the first years of professional practice. Considerable clinical experience is necessary for medical students and young practitioners in their learning process. Young practitioners need to be more included and to be participants in the professional community. It seems important to stimulate more systematically to critical reflection on clinical experiences and to deliver more performance feedback to medical students and young practitioners. More attention to emotions and emotional reactions seems to be needed in the learning process.

## Conclusion

This study provides additional information about the lived learning experiences of young physicians dealing with suicidal patients during the critical transition phase from medical school to their early professional life. The informants gave a detailed account of the learning process; from recognising similarities and differences in patients they have treated, to accumulating pattern knowledge, which then contributed to their personal feelings of competence and confidence. They described their personal competence with cognitive and emotional elements consisting of both articulated and less articulated knowledge. Peers are used for both learning and support and might represent a more powerful resource in the learning process than previously acknowledged. Emotions do not seem to be adequately focused upon in supervision, which is important both from a learning perspective and for the well-being of young professionals. The study indicates that there are fields with greater potential for more extensive and comprehensive learning. These include stimulating more systematically to critical reflection on and in practice, attention to feelings in the reflective process and provision of more performance feedback to young physicians.

## Competing interests

The author(s) declare that they have no competing interests.

## Authors' contributions

TSH and RO have conceived the study, TSH carried out the interviews, done the analysis and written the draft of the manuscript. AGT contributed substantially in the process of design and analysis and AGT and RO have critically revised the manuscript. All three authors have read the manuscript and approved it for publication (Vancouver Convention).

## Pre-publication history

The pre-publication history for this paper can be accessed here:



## Supplementary Material

Additional file 1 Interview guide: Experiences from treating suicidal patients. The document is the semi-structured interview guide used for the qualitative interviews Click here for file
