# Supplementary material for: A qualitative study of the learning processes in young physicians treating suicidal patients: from insecurity to personal pattern knowledge and self-confidence
Source: BMC Med Educ. 2007 Jul 6;7:21. doi: 10.1186/1472-6920-7-21 (PMC1950702; doi:10.1186/1472-6920-7-21)
Supplement: Additional file 1 — Interview guide: Experiences from treating suicidal patients. The document is the semi-structured interview guide used for the qualitative interviews [file 1472-6920-7-21-S1.doc]

# Interview guide, final version

# Tordis Sørensen Høifødt

**Experiences from treating suicidal patients**

What has been important for you?

In what way has it been important?

1. **The story of treating a suicidal patient**

**How did you think?**

**How did you feel?**

**How did you do?**

How did this element have impact on you in meeting the patient?

How did it have impact on your patient?

What kind of feelings did this touch in you?

How did you deal with your feelings in this situation?

In what way did your feelings affect your experience of the patient?

How did you deal with the patient’s feelings?

How did your feels affect the meeting with the patient and the development of the encounter?

1. **Reflection on the clinical situation from a perspective of learning?**
   - When we have gone through this story, did you get any new thoughts so that you look upon the situation from a new perspective?
   - When you look back, was there any kind of preparation ahead of time that could have been useful?

How did your knowledge have impact on you in this situation?

How did your skills have impact on you in this situation?

How were you affected of by your own attitudes to suicide?

- What are your attitudes to suicide?
- How were you affected by your attitudes?
- How do your attitudes affect your patient?
- How do your attitudes affect the encounter with the patient?

How do your colleagues´ attitudes to suicidal patients have impact on you?

How could you have dealt with this situation differently?

Did you feel that you lacked knowledge to handle this situation?

Did you receive any supervision?

Do you have experience with supervision?

Did you benefit from supervision? In what way?

How do you consider your own confidence?

What is confidence for you?

How does your confidence affect your thoughts, feelings and behaviour?

Have any teaching experiences in medical school or reading literature been of importance? How?

Did any other experiences in life in general affect you in meeting this clinical situation? How?
